# Correlation analysis of upper limb muscle activation in the frequency domain in wheelchair fencers

**DOI:** 10.3389/fnhum.2025.1523358

**Published:** 2025-02-25

**Authors:** Monika Błaszczyszyn, Katarzyna Piechota, Zbigniew Borysiuk, Krzysztof Kręcisz, Dariusz Zmarzły

**Affiliations:** ^1^Faculty of Physical Education and Physiotherapy, Opole University of Technology, Opole, Poland; ^2^Faculty of Electrical Engineering, Automatics and Computer Science, Opole University of Technology, Opole, Poland

**Keywords:** intermuscular synchronization, disability sports, wavelet analysis, cross-correlation, frequency bands

## Abstract

**Background:**

The study includes a correlation analysis of EMG signals of upper limb muscle activity in wheelchair fencers. The aim of the study was to investigate neuromuscular conduction in wheelchair fencers using the EMG signal from their upper limb muscles.

**Methods:**

Wavelet transform analysis was used to examine the biosignals. The recorded EMG signals were subjected to time-frequency transformations. The scalograms were determined using the continuous wavelet transform. Based on the analysis, time-frequency coherence maps were extracted to determine validation in the frequency bands: 2–16 Hz, 17–30 Hz, and 31–60 Hz. The study participants were 16 wheelchair fencers, members of the Polish Paralympic Team, in two disability categories: 7 in category A and 9 in category B. Coherence was calculated for frequencies up to 60 Hz.

**Results:**

The analysis revealed the individual time-dependent coherence between two signals for different frequencies during the work cycle of the antagonist muscles of the arm (biceps/triceps) and forearm (flexor/extensor carpi radialis). A significant difference in alpha coherence (2–16 Hz) occurred in the group of forearm muscles in the frequency band of 2–16 Hz, both for G (*p* = 0.042) and M (*p* = 0.031) parameters (G: A - 0.08 Hz, B - 0.04 Hz; M: A - 0.51 and B - 0.42). Its peaks were observed during the fencing action cycle. Some differences in gamma coherence were also found in the EMG signals of the forearm muscles in the 31–60 Hz frequency band were statistically significant (*p* = 0.031): 0.43 in group A and 0.36 in group B.

**Conclusion:**

The results showed the neuromuscular conduction, where alpha coherence reflects the reticulospinal tract responsible for the excitation of the distal muscles of the wrist and hand, while gamma coherence results from cortical signals. It is related to efferent conduction and reflects corticomuscular coupling. Frequency domain coherence analysis determines the strength of intermuscular synchronization, allowing a comprehensive investigation of the neural mechanisms underlying motor recovery. It maps separate neural pathways for arm and hand control.

## 1 Introduction

Surface electromyography (sEMG) is one of the most commonly used measures of muscle activation. The methods of biosignal processing and analysis, such as: intermuscular coherence (IMC), Continuous Wavelet Transform (CWT), Cross-correlation offer increasingly accurate ways to monitor individual components of the recorded signal and provide insightful analysis of working muscles during motor activities (Kenville et al., 2020). While EMG allows the characterization of working muscles, intermuscular coherence and synchronization analysis of the EMG signal can explain the phenomenon of neuromuscular conduction during motor activity (d'Avella et al., 2003; Mohr et al., 2018). EMG detects the electrical potentials generated by muscle cells when activated. The motor unit action potential is a compound potential representing the sum of the individual action potentials. Motor units must synchronize their connections in order to tone the entire muscle smoothly, which requires common presynaptic stimulation of motor neurons. A popular method for testing motor control is intermuscular coherence (IMC). Using IMC, it is possible to non-invasively study the common synaptic signal for pools of motor neurons in muscles (Gross et al., 2002; Dideriksen et al., 2018). Any recorded signal, including EMG, can be viewed as the sum of determined and random components. The former carry information about the recorded signal. The latter contain information about noise and artifacts during signal recording. To separate the signal, a wavelet transform is used, which consists of selecting the wavelet that is most similar to the recorded signal, called the mother wavelet. This results in the separation of parts of the signal that are similar to the selected pattern. The operation of a wavelet is similar to that of a low-pass filter. The wavelet transform involves the decomposition of the signal into the approximation and detail coefficients. The approximation represents the component that carries spectral diagnostic information. The result is a frequency spectrum map (Ngui et al., 2013; Ghofrani Jahromi et al., 2017).

In the present study, the Continuous Wavelet Transform (CWT) method was used. The CWT effectively identifies linear coupling between EMG signals during different movement sequences. This method provides information about the level and strength of coupling in the form of time-frequency maps. On this basis, the associated characteristics of the signals in specific frequency bands can be visualized (Garg et al., 2013; Rocha et al., 2022). One of the most reliable techniques for assessing the level of motor unit synchronization in human muscles is the cross-correlation of the discharge times of two simultaneously active motor units. Cross-correlation has become the most widely used method for determining the interdependence of human motor unit discharges (Semmler, 2002). The frequency domain equivalent of cross-correlation analysis has been used in human motor unit studies to detect periodic triggering of common synaptic input signals to motor neurons. Studies have shown that coherence can be detected between pairs of motor units in the hand muscle in frequency bands of 1–12 Hz and 16–32 Hz during voluntary isometric abduction of the index finger. The observed significant coherence between pairs of motor units at these frequencies implies a common periodicity of the presynaptic signal (Farmer et al., 1993; Raikova et al., 2021). Commonly studied frequency bands include the alpha (8–15 Hz), beta (15–30 Hz), and low gamma (30–60 Hz) bands, as coherence within these bands is thought to originate from different neural sources (Kim et al., 2001). These bands are generally used to represent common neuronal inputs associated with corticospinal pathways (Weersink et al., 2021). The coherence of the alpha band originates subcortically, and the alpha band is thought to specifically reflect the involvement of the reticulospinal tract (Laine et al., 2021).

The coherence of the alpha band is related to the degree to which the overall level of muscle activity is correlated over time (Grosse and Brown, 2003). In contrast, the beta band is associated with corticospinal conduction and reflects the synchronization between the motor cortex and the working muscles. Intermuscular coherence between flexor and extensor muscle pairs is mainly observed in the beta band (Hu et al., 2018). Intermuscular coherence in the gamma band is due to cortical signals and is thought to be of functional importance in efferent conduction (Farmer et al., 1998).

The aim of the study was to investigate neuromuscular conduction in wheelchair fencers using the EMG signal from their upper limb muscles. For this purpose, the bands (1) 2–16 Hz, (2) 17–30 Hz, and (3) 31–60 Hz were selected to assess the activation of opposite muscle groups of the upper extremities: the biceps/triceps for arm movement and the flexor carpi radialis/extensor carpi radialis for forearm movement.

## 2 Methods

### 2.1 Ethical approval

The study included an analysis of the electromyographic signal for upper limb movement during fencing performance. The study was approved by the Bioethics Committee of the Medical Chamber (Resolution No. 237 of December 13, 2016) in accordance with the Declaration of Helsinki. Each participant gave written informed consent to participate in the study, after both verbal and written explanations of the study procedures.

### 2.2 Participants

Wheelchair fencing athletes are classified into three categories (A, B, C) with respect to their movement capacity. Only categories A and B feature at the Paralympic Games, athletes in category C can also compete, as these athletes have the most severe impairments are currently least represented in the sport. Category A comprises fencers after lower limbs amputation or with partial paresis, having freedom of movement of the trunk and arms. Category B includes athletes with weakened trunk stabilization caused by, e.g., paraplegia (transverse paraplegia) with a ruptured spinal cord, paralyzed lower limbs and/or minimal hand paresis (Fletcher et al., 2021; Borysiuk et al., 2022).

Sixteen members of the Polish Paralympic Wheelchair Fencing Team were selected to participate in the study. Detailed characteristics of each group are provided in Table 1. All participants were right-handed.

**Table 1:** Demographical and clinical features of wheelchairs fencers in categories A and B.

Category (N)	Age (years), mean ± SD	Height (m), mean ± SD	Mass (kg), mean ± SD	Training (years), mean ± SD	Neurological disease	No-neurological disease
A (7)	32.57 ± 6.25	1.74 ± 0.09	69 ± 8.93	14.29 ± 6.32	5	2
B (9)	30.33 ± 9.67	1.64 ± 0.11	58.67 ± 10.3	6 ± 2.4	9	0

### 2.3 EMG instrumentation

EMG signals were recorded using a 16-channel EMG system (Noraxon, DTS, Desktop Direct Transmission System, Scottsdale, Arizona, USA) with a 16-bit sampling rate of 1,500 Hz. Dedicated software (MyoResearch XP Master Edition for DTS Noraxon) was used to analyze the system data. The raw EMG signal was processed using zero-mean normalization. The root mean square (RMS) method was used to extract features. Paired bipolar Ag-AgCl EMG surface electrodes were placed bilaterally on four muscles: the flexor carpi radialis/extensor carpi radialis of the forearm and the biceps/triceps of the arm. EMG electrodes were placed according to the SENIAM guidelines (www.seniam.org, date of access 11.09.2021), i.e., with bipolar pairs oriented parallel to the muscle fibers with a distance of 20 mm between electrodes. Measurement procedures using EMG have been described in detail in previous studies (Borysiuk et al., 2020a; Błaszczyszyn et al., 2021). 1. Taking a fencing position on a wheelchair, the wheelchair attached to a special frame for stabilization. 2. Measuring the distance between the athletes or the athlete and the coach. 3. Preparing the athletes for testing: skin preparation, electrode attachment. 4. Individual warm-up with the coach for 20–25 min. 5. Testing - only athletes classify as category A and B took part in this study. During the tests, the fencers used an épée with a weight of 750 g. The surface hit by the weapon included the torso and arms and a head mask. Throughout the test procedure, the coach initiated three series of simple lunge attacks in response to a visual stimulus (Borysiuk et al., 2020b).

### 2.4 EMG data pre-processing and analysis

Research methodology: (i) raw EMG signal, (ii) normalization and evaluation, (iii) wavelet coherence analysis, (iv) cross-correlation.

A correlation analysis of recorded upper limb EMG signals in wheelchair fencers was performed. The study used wavelet transform analysis to analyze the biosignals (Rafiee et al., 2011). The Daubechies wavelet, a wavelet with a continuous compact carrier, was chosen as the mother wavelet. It is a wavelet perfectly localized in time. It is characterized by an accurate approximation of the function and a relatively simple form, most similar to the physiological signal from human muscles. The recorded EMG signals were subjected to time-frequency transformation. The scalograms were determined using the CWT. In order to assess the level of the obtained correlations of the muscles of the arm (biceps/triceps) and forearm (flexor/extensor carpi radialis), wavelet coherence waveforms were calculated in the time and frequency domain, and time-frequency coherence maps were obtained for validation in frequency bands (2–16 Hz, 17–30 Hz, and 31–60 Hz). The following signal sets were derived from the bandpass-filtered signal as described in the literature (Bonato et al., 2001; Klein et al., 2006; Nazarpour et al., 2013).

### 2.5 Statistical methods

Statistical analysis was performed using Statistica 13.1 (StatSoft, Inc., Oklahoma, USA). Nonparametric tests were used to detect abnormal data distributions. The Kruskall-Wallis test was used to compare different controlled muscle activation protocols in the first cohort. The Mann–Whitney U test was used to compare the intramuscular coherence of trunk muscles between category A and category B wheelchair fencers. The level of statistical significance was set at *p* ≤ 0.05.

To determine the differences between fencer groups A and B, coherence was evaluated for the antagonistic muscles of the upper limb: biceps/triceps and flexor/extensor carpi radialis. The analysis included three frequency bands for which the following parameters were calculated: gband (G)—the proportion of high coherence (above 0.9) in the total coherence in a given frequency band; and mband (M)—the mean coherence in a given frequency band, calculated from the total movement time (Torrence and Webster, 1999; Grinsted et al., 2004).

## 3 Results

### 3.1 Wavelet coherence estimation in the time domain

**Figure 1:**
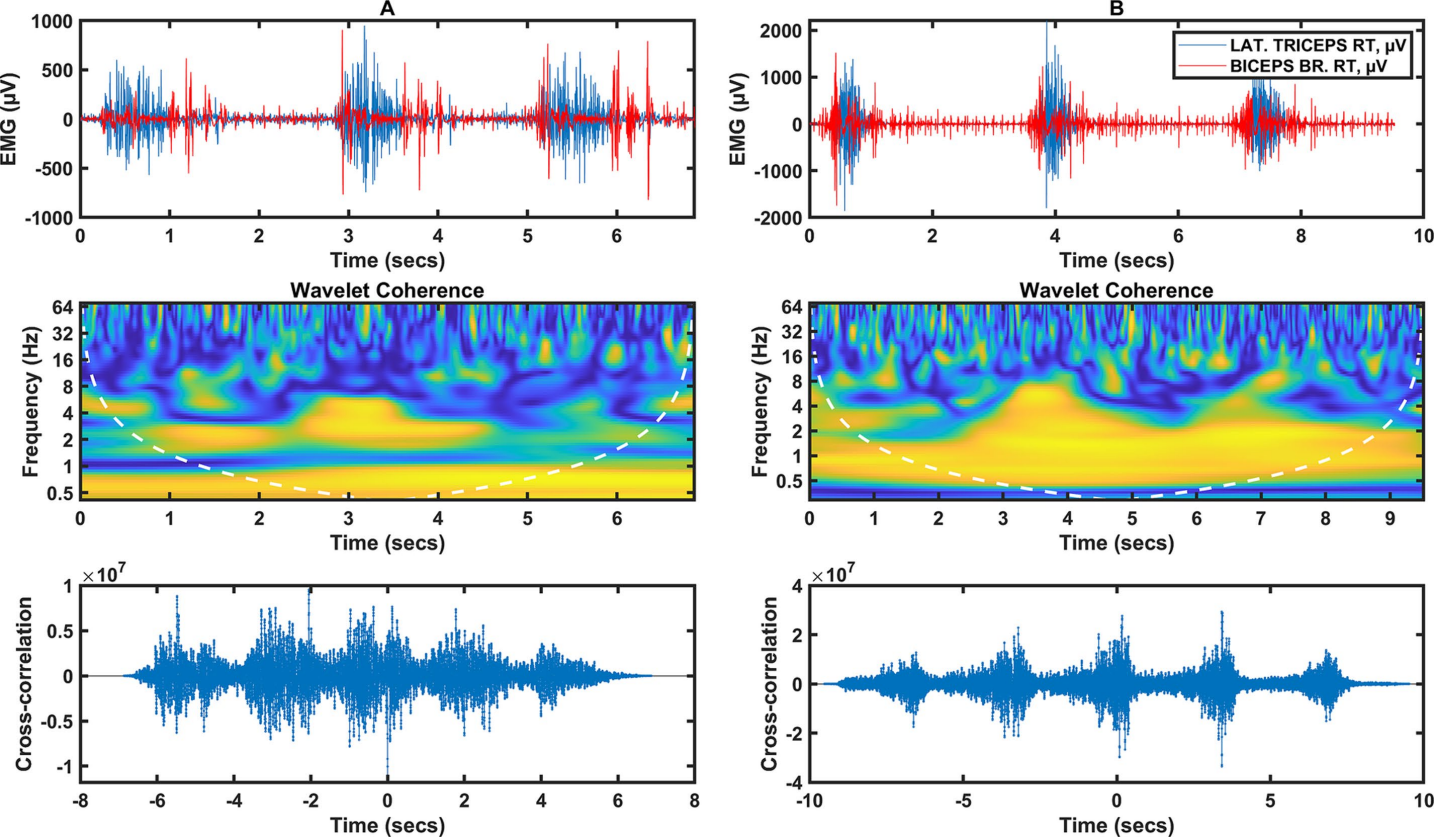
Two columns of data illustrating the arm muscle signal (triceps/biceps). Column **A** represents data from category A fencers, column **B** from category B fencers. Row 1 represents the raw EMG of the shoulder muscles during the fencing actions, the peaks of muscle activity indicate the timing of the fencing action (3 actions). Row 2 shows the wavelet coherence of selected muscles in the selected frequency bands. Row 3 shows the cross-coherence between muscle groups.

**Figure 2:**
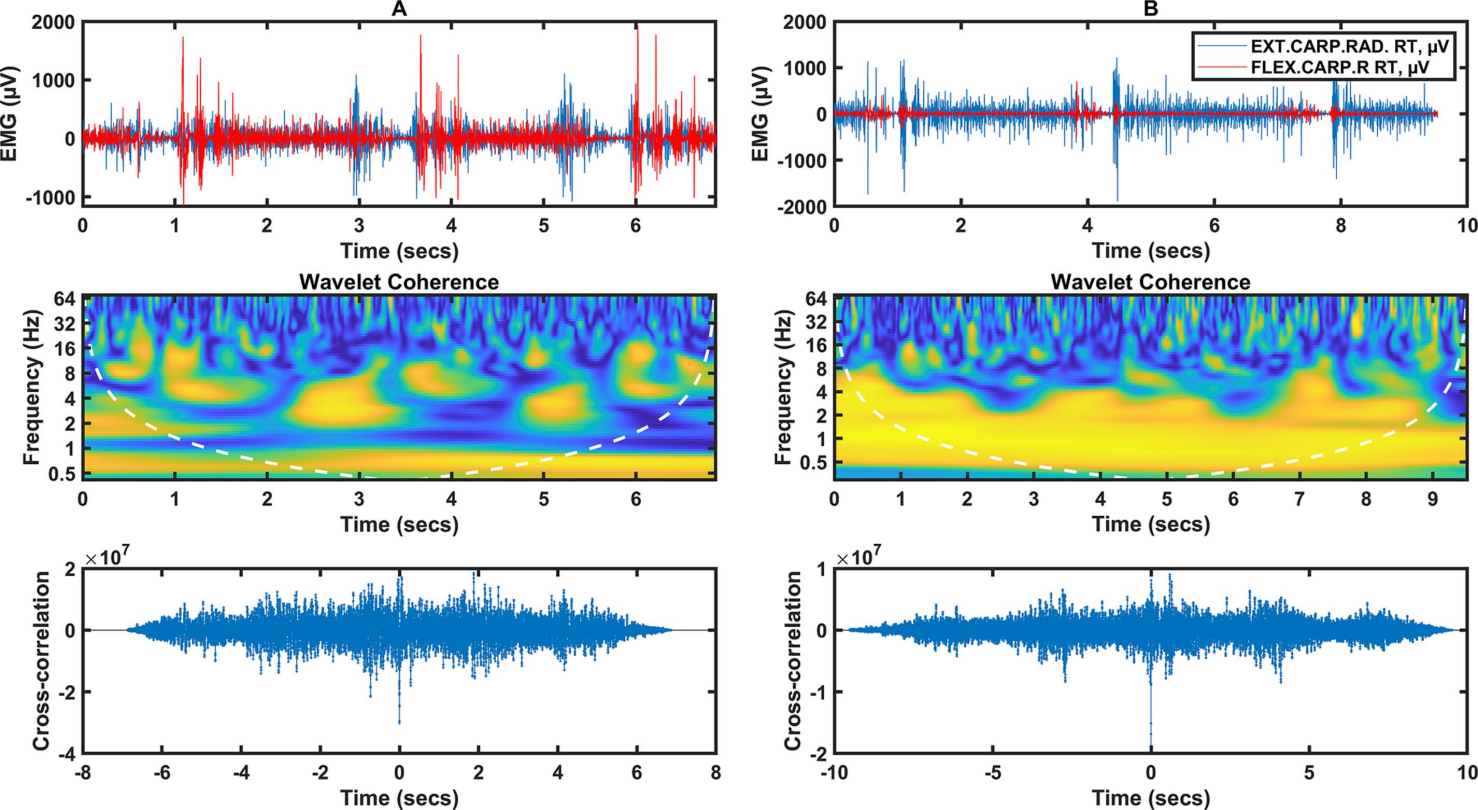
Two columns of data illustrating the forearm muscle signal (flexor/extensor carpi radialis). Column **A** represents data from category A fencers, column **B** from category B fencers. Row 1 shows the raw EMG during the fencing actions, the peaks of muscle activity indicate the timing of the fencing action (3 actions). Row 2 shows the wavelet coherence of selected muscles in the selected frequency bands. Row 3 shows the cross-coherence between muscle groups.

The coherence between the selected muscles can be transformed into the time domain (Figures 1, 2) to show whether the coherent signals from the arm and forearm muscles are fully synchronized or whether one signal precedes or follows the other. These time domain estimates can be separated into three components. First, if the signals are fully synchronized in the time domain, a peak of approximately 0 ms is observed, indicating joint neuromuscular control. Second, signals from antagonistic muscles were shown in the time domain estimates as significant peaks with positive time lags. Positive time delays can be divided into intervals corresponding to the conduction times of subcortical or cortical pathways.

For category A fencers (Figure 1A), there is a correlation in the frequency domain in the very low frequency range below 1 Hz over the entire time interval from 0 to 7 s, which is probably not so much related to the EMG activity, but rather due to the distance between the activities. Similarly, in Figure 1B, the frequencies in the 1 Hz range are due to the interrelationship between whole activities. For category A, there is synchronization between activities for 2 Hz frequencies in the 1 to 2 s and 3 to 4 s time intervals. There is also synchronization for 4 Hz in the 0 to 0.5 s and 3 to 3.5 s time intervals. There is no significant coherence between 8 and 64 Hz.

For category B fencers (Figure 1B), there is a significant correlation for frequencies of 0.5 to 2 Hz over the entire time interval analyzed. In the 2 to 8 Hz range, there is coherence for activity at 3 to 4 s and 6 to 7 s. This is consistent with the time course for which muscle activity events occur simultaneously and are synchronized in the low frequency band.

For category A fencers (Figure 2A), wavelet coherence for frequencies below 1 Hz occurs in the time interval from 0 to 7 Hz, independent of the activity of individual muscles, but dependent on the temporal relationship between fencing actions. Coherence occurs from 2 to 16 Hz. There is the strongest short-term synchronization of a few 100 ms at 2.5 and 5 s in the 3 Hz band; strong synchronization is also found at frequencies of 6–8 Hz, in the form of short-term moments of synchronization of wavelet components.

A different coherence distribution can be observed for category B fencers (Figure 2B), where there is a strong coherence in the range of 0.5 to 4 Hz throughout the analyzed time interval. This is also confirmed by the time course, where a high correspondence and synchronization of the EMG waveforms in both channels can be observed for EXT CARP RAD and FLEX CARP R muscles.

### 3.2 Statistical analysis

#### 3.2.1 TRICEPS vs. BICEPS

**Figure 3:**
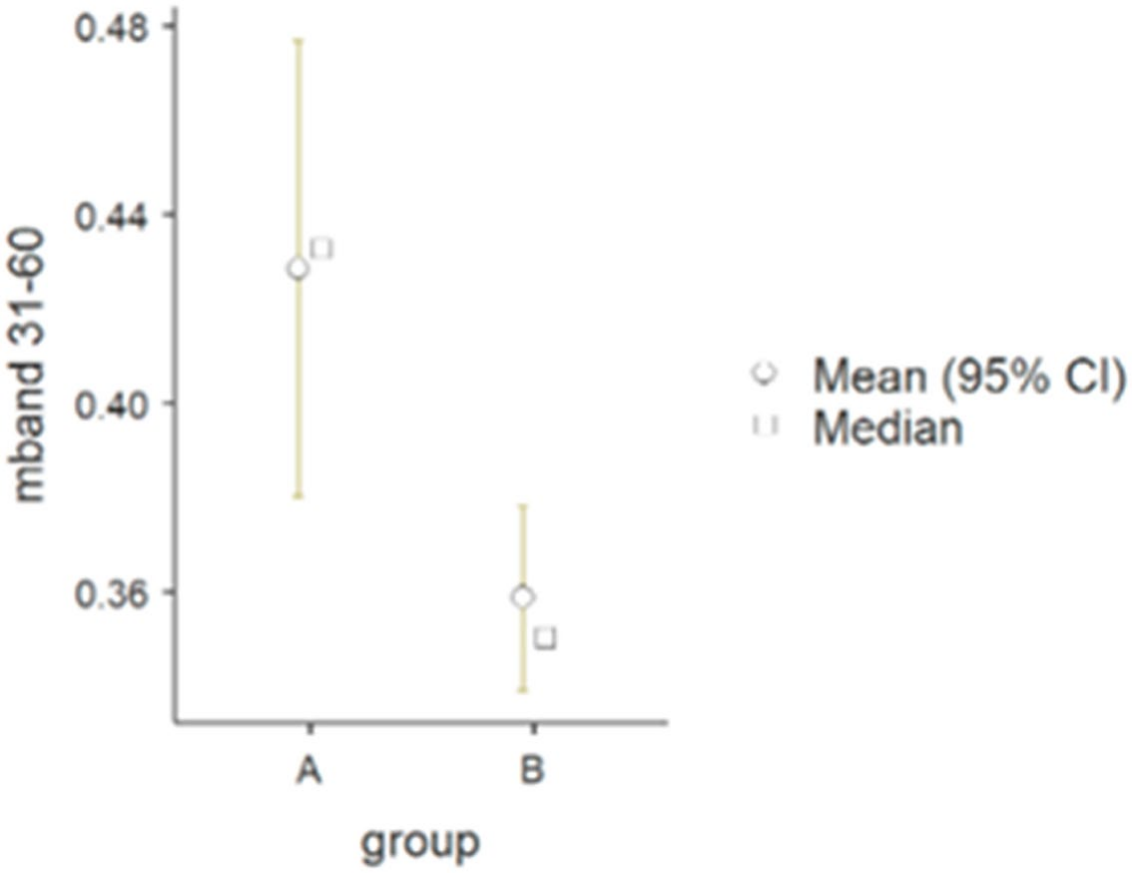
Differences between wheelchair fencers from groups A and B for the pair of arm muscles in the 31–60 Hz frequency band (mband) for parameter M.

The results of the Mann–Whitney U-test (Table 2) for the arm muscle groups (triceps/biceps) in the 31–60 Hz frequency band were statistically significant (*p* = 0.031): 0.43 in group A and 0.36 in group B (see Figure 3).

**Table 2:** Mann–Whitney U test results for arm muscle groups (triceps vs. biceps) in wheelchair fencers (categories A and B).

Band	Test-U	Statistic	*p*	Correlation	Effect size
gband 2–16	Mann–Whitney U	29.00	0.837	Rank biserial correlation	0.08
gband 17–30	Mann–Whitney U	20.00	0.252	Rank biserial correlation	0.37
gband 31–60	Mann–Whitney U	13.00	0.055	Rank biserial correlation	0.59
mband 2–16	Mann–Whitney U	31.00	1.000	Rank biserial correlation	0.02
mband 17–30	Mann–Whitney U	24.00	0.470	Rank biserial correlation	0.24
mband 31–60	Mann–Whitney U	11.00	0.031	Rank biserial correlation	0.65
maxlag	Mann–Whitney U	29.00	0.815	Rank biserial correlation	0.08

#### 3.2.2 Flexor carpi radialis (FCR) vs. extensor carpi radialis (ECR)

**Figure 4:**
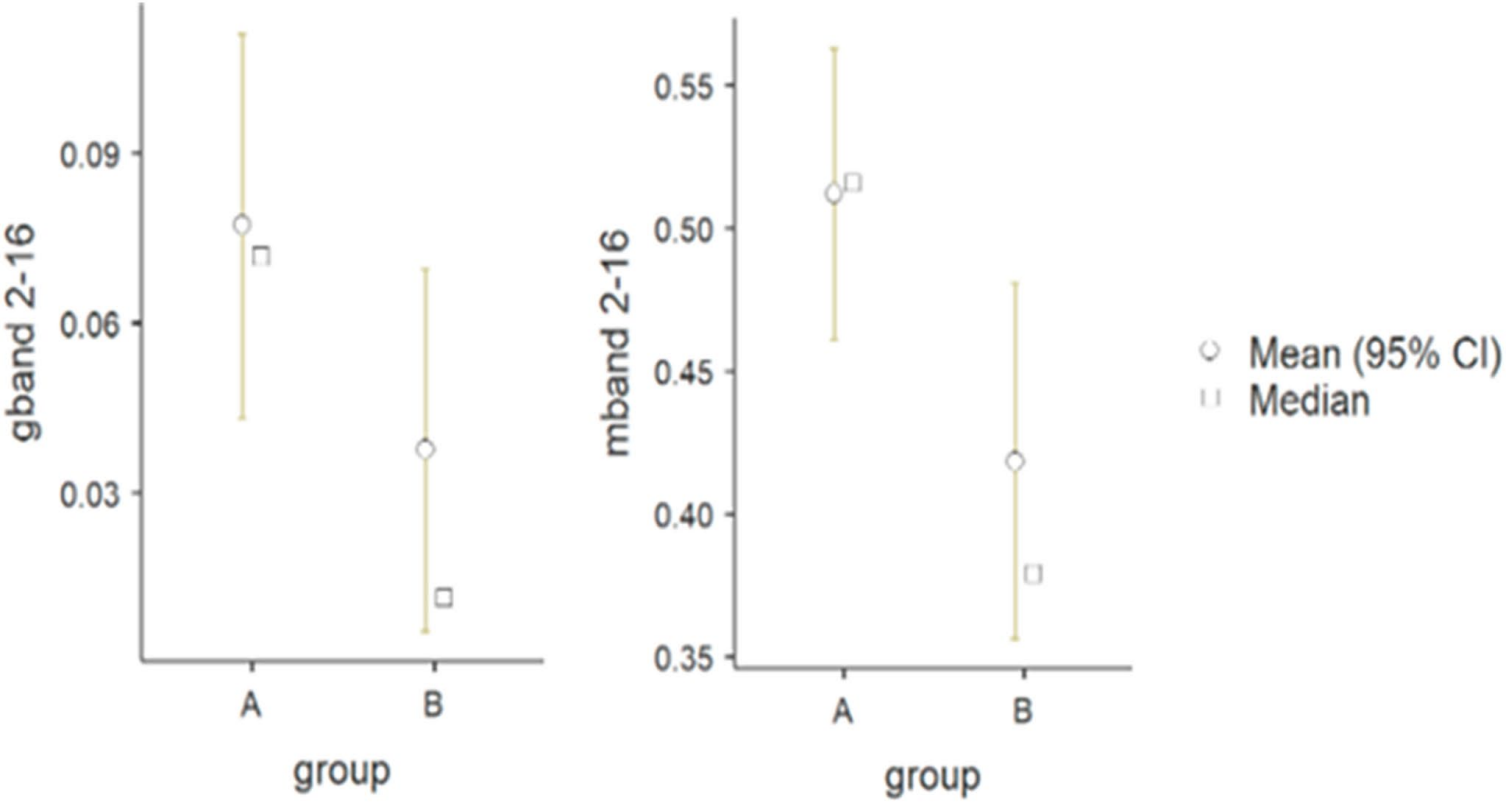
Differences between wheelchair fencers of groups A and B for the pair of forearm muscles in the 2–16 Hz frequency band for the M (mband) and G (gband) parameters.

There was statistical significance in the forearm muscle group (flexor vs. extensor carpi radialis) in the frequency band of 2–16 Hz, both for G (*p* = 0.042) and M (*p* = 0.031) parameters (G: A - 0.08 Hz, B - 0.04 Hz; M: A - 0.51 and B - 0.42) (see Figure 4).

## 4 Discussion

The purpose of comparing category A and category B fencers was to explore how varying levels of muscle activation (resulting from differences in impairments) affect performance in fencing. Category A athletes, who generally experience less severe impairments, are able to activate muscles involved in fencing more fully and may demonstrate muscle patterns more typical of able-bodied athletes. On the other hand, category B athletes often have limited or altered muscle activation due to neurological or musculoskeletal conditions, which may lead to adaptive strategies in how they control their weapons and execute movements. The procedure used resulted in a normalized measure of correlation in the frequency domain, ranging from 0 to 1. Coherence was calculated for frequencies up to 60 Hz. The analysis illustrates the individual time-dependent coherence between two signals at different frequencies during the muscle work cycle. A statistically significant difference in alpha coherence (2–16 Hz) was found in the forearm muscle group (Table 3), and its peaks were observed during periods of the fencing action cycle (Figure 2). Some differences were also found in the gamma coherence of the arm muscle signals (Table 2). Conversely, no significant activity was observed in the beta frequency band. Motor unit synchronization in the beta (15–30 Hz) and low gamma (30–60 Hz) bands was mainly driven by the primary motor cortex and less affected by the premotor cortices and supplementary motor area (Marsden et al., 2001; Chen et al., 2018; Obata et al., 2014). The results confirm the line with the other studies in which alpha reflects the reticulospinal tract, responsible for stimulation of the distal muscles of the wrist and hand, while gamma is derived from cortical signals and is related to efferent conduction and reflects corticomuscular coupling. The reticulospinal tract modulates muscle tension and reflex activity. It also causes reciprocal inhibition of antagonistic muscles. According to current knowledge, muscular synchronization can be altered by the loss or reduction of correlated neuronal inputs from the central nervous system; in such cases, motor units typically operate at a frequency of 5 to 12 Hz, indicating that there is a correlation between the oscillation generated in the CNS and the firing rate of the motor unit (Person and Kudina, 1972).

**Table 3:** The Mann–Whitney U test results for forearm muscle groups (flexor vs. extensor carpi radialis) in wheelchair fencers (categories A and B).

Band	Test-U	Statistic	*p*	Correlation	Effect size
gband 2–16	Mann–Whitney U	12.00	0.042	Rank biserial correlation	0.62
gband 17–30	Mann–Whitney U	23.00	0.408	Rank biserial correlation	0.27
gband 31–60	Mann–Whitney U	18.00	0.174	Rank biserial correlation	0.43
mband 2–16	Mann–Whitney U	11.00	0.031	Rank biserial correlation	0.65
mband 17–30	Mann–Whitney U	20.00	0.252	Rank biserial correlation	0.37
mband 31–60	Mann–Whitney U	20.00	0.252	Rank biserial correlation	0.37
maxlag	Mann–Whitney U	26.50	0.622	Rank biserial correlation	0.16

Irregularities in cortical control are responsible for the loss of alpha conduction in individuals with muscle paralysis and hemiparesis. The existence of coherence between low-gamma muscles can be explained by the need to quickly integrate information under dynamic conditions and generate appropriate motor commands. It reflects conduction between cortical activity and working muscles. The corticoreticulospinal tract (CReST) is a major descending motor pathway in many animals, but little is known about its innervation patterns in proximal and distal upper extremity muscles in humans (Taga et al., 2021). Recent studies conducted in monkeys have shown that the reticulospinal projections innervate the intrinsic muscles of the hand, with the strength of the wrist and hand muscles rivaling that of the more proximal muscles. The gamma, on the other hand, is associated with efferent conduction. In recent years, muscle synergy analysis has been widely used to determine how muscles are coordinated during complex motor activities. Results have shown that children with spastic cerebral palsy activate fewer synergies, for example in gait patterns, than children without neurological deficits (damage) (Goudriaan et al., 2022). Kisiel-Sajewicz et al. (2011), reported that people with limbic paralysis showed weaker intermuscular coupling in the low-frequency range (0–11 Hz), but these were stroke patients with movement problems. In contrast, Fisher et al. found that IMC in the beta band (15–30 Hz) can be used as a biomarker to assess upper limb motor dysfunction in stroke patients (Fisher et al., 2012). In a study of athletes with disabilities, IMC was obtained in low frequency bands. Despite the above findings, one of the main limitations of muscle synergy analysis is that it can only reflect the spatiotemporal anatomical coordination of multiple muscles and assess the activation state of overall muscle activity (Liu et al., 2022), but it does not reveal the neurophysiological mechanisms underlying the formation of muscle synergies (d'Avella et al., 2003). Coherence analysis is an effective method for assessing neural synchronization between muscles or brain areas and is usually based on cross-correlation between signals in the frequency domain (Li et al., 2016).

The human upper limb is capable of performing a variety of functional tasks due to its biomechanical structure and abundant motor and sensory innervation. Previous studies of the human arm have shown altered kinematics of the hemiparetic arm and a reduced ability to generate the required torques in upper limb joint interactions (Cirstea et al., 2003). Abnormal neurological coupling as part of flexion synergy between the elbow and shoulder muscles has also been described after stroke (Ellis et al., 2017). These synergies have also been shown to extend to the finger flexors. Indeed, the number of distinct activation patterns appears to be reduced after nervous system damage (McPherson and Dewald, 2019). However, most of the studies were based on the evaluation of patients with motor limitations. The present study is based on the performance of highly trained Paralympic athletes. It is worth noting that neural synchronization has been recognized as a mechanism that integrates the distributed sensory and motor systems involved in the coordination of complex movements. Muscle synergy represents the anatomical coordination of muscle activation over time during the performance of a specific motor task. This means that oscillatory coupling and synchronous neuronal discharges play an important role in the process of human movement control (d'Avella et al., 2003). It was Bernstein who first proposed that the central nervous system would be able to jointly activate a group of muscles to overcome the “many degrees of freedom” problem in motor control (Bernstein, 1966). D'Avella et al. (2003), proposed that the activation of interacting muscle groups is coordinated by descending correlated neural excitations. Studies from the perspective of neural oscillations conveying motor control information have suggested that IMC may reflect correlated intermuscular neural inputs in healthy subjects (Fisher et al., 2012; De Marchis et al., 2015; Hu et al., 2018). Although existing literatures cover functionalities of muscle network or synergy patterns separately, little evidence shows their collective mechanism. Luo et al. (2024) deciphered the mechanism of synergy patterns on muscle network among lower-limb muscles. Individuals exhibiting notable deficiencies in their synergy patterns should initially engage in targeted exercises to reconstruct patterns within the local muscle network before extending the approach to the global network to fully reinstate motor abilities.

Increased neural coupling between distal and proximal muscles can affect the basic modular structures of the spinal cord that control arm and hand muscles during functional activities. Manipulating objects, a common part of performing a task, requires further coordination between the joints as the arm must position, orient, and stabilize the hand while the fingers interact with an external object. Separate neural pathways for arm and hand control have been proposed, and the extent of communication between these pathways remains unclear (Phan et al., 2022). The results of the present study suggest that the increased level of hand muscle activation at target locations requiring arm abduction and elbow extension can be explained by increased neural coupling between the forearm (flexor/extensor carpi radialis) and arm muscles (biceps/triceps) (Li et al., 2016). In the synchronization of the arm muscles, for category A wheelchair fencers (Figure 1A), there was synchronization between the activities for the 2 Hz frequency in the time intervals from 1 to 2 s and from 3 to 4 s, as well as for the 4 Hz frequency in the time intervals from 0 to 0.5 s and from 3 to 3.5 s. On the other hand, for the forearm muscles of category A wheelchair fencers (Figure 2A), coherence occurred in the range from 2 to 16 Hz, in the form of short-term discharges (with a duration of several hundred ms) at 2.5, 5 s in the 3 Hz band. There was also strong synchronization at 6–8 Hz in the form of brief moments of synchronization of wavelet components. For the category B wheelchair fencers (Figure 1B), synchronization of the arm muscles occurred in the 2 to 8 Hz range at 3 to 4 s and 6 to 7 s. This is consistent with a time course where muscle activity events occur simultaneously and are synchronized in the low frequency range. A different coherence distribution is observed for the forearm muscles of category B wheelchair fencers (Figure 2B), where strong coherence was found in the 0.5 to 4 Hz range over the entire analyzed time interval. The observed coherence over the whole analyzed interval within the forearm muscles in the group of category B fencers reflects the effect of integration with the object, i.e., the continuous holding of the weapon by the fencer. Such an effect is not observed in category A fencers, in whom distinct periods of rest and stimulation can be observed. In addition, category B wheelchair fencers show a high consistency and synchronization of EMG waveforms in both channels mapped for the forearm muscles.

## 5 Conclusion

The intermuscular coherence analysis revealed that homologous muscle functions are induced by common oscillatory inputs that include alpha, beta, and gamma frequencies with different synchronization patterns during different movement periods. The results of the study confirm the existence of different oscillations in the alpha and gamma frequency bands depending on motor performance and the degree of neuromuscular conduction impairment (category A and B wheelchair fencers), which explains the theory of alpha band excitation, characteristic of subcortical conduction, that reticulospinal pathways modulate muscle tension and reflex activity. They also cause reciprocal inhibition of antagonistic muscles. This is especially true in precision movements performed with forearm muscles. Unlike the gamma band, which reflects cortical conduction, corticospinal pathways carry muscle control impulses from the cortex through the spinal cord to spinal nerves and muscles, providing stability especially during fast and precise motor actions.

## Data Availability

The raw data supporting the conclusions of this article will be made available by the authors, without undue reservation.
